# Supplementary material for: A novel method to test non-exclusive hypotheses applied to Arctic ice projections from dependent models
Source: Nat Commun. 2019 Jul 9;10:3016. doi: 10.1038/s41467-019-10561-x (PMC6616623; doi:10.1038/s41467-019-10561-x)
Supplement: Supplementary file 1 — Supplementary Information [file 41467_2019_10561_MOESM1_ESM.pdf]

Supplementary Information

A novel method to test non-exclusive hypotheses applied to Arctic ice projections from dependent models

R. Olson et al.

Supplementary Figures

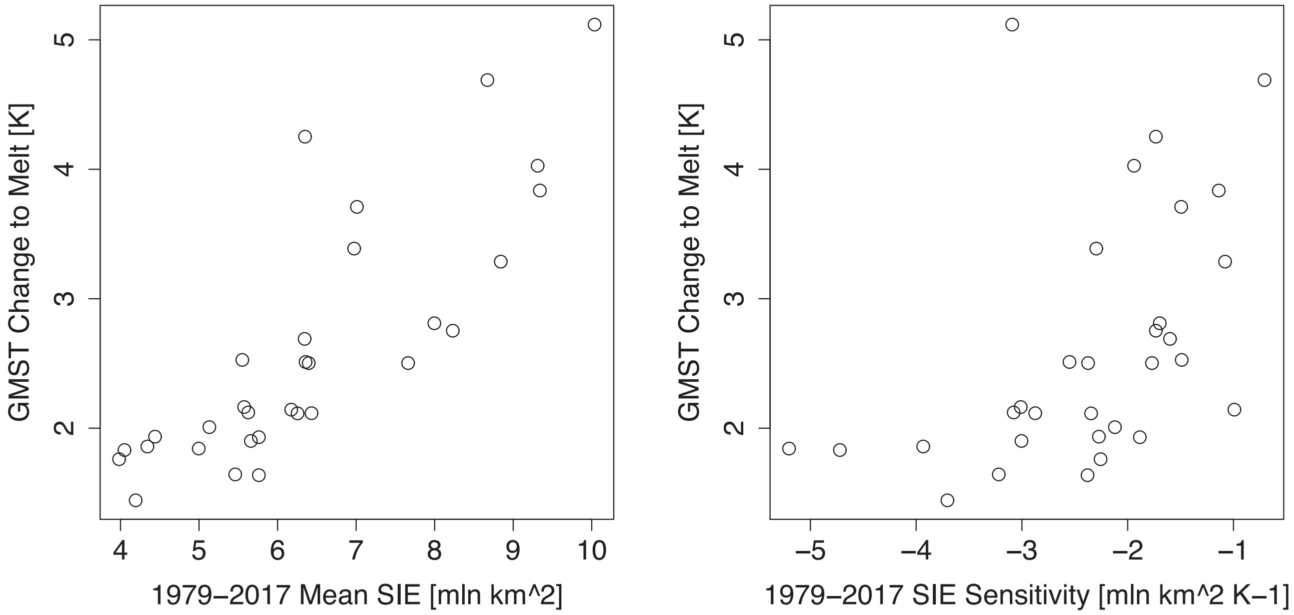

Supplementary Figure 1: **Relationship between present-day and future sea ice metrics.** Scatter plots between (left) mean 1979-2017 sea ice extent (SIE) [million km^2] and global mean temperature (GMST) change needed for September Arctic sea ice melt ("GMST change to melt") [K] in the climate models we use, and (right) 1979-2017 SIE sensitivity to GMST [million km^2 K^{-1}] and GMST change to melt [K]. This figure illustrates the relationships between SIE and its sensitivity to temperature, and the GMST change to melt. These relationships motivate us to use these present-day SIE metrics as constraints on climate models.

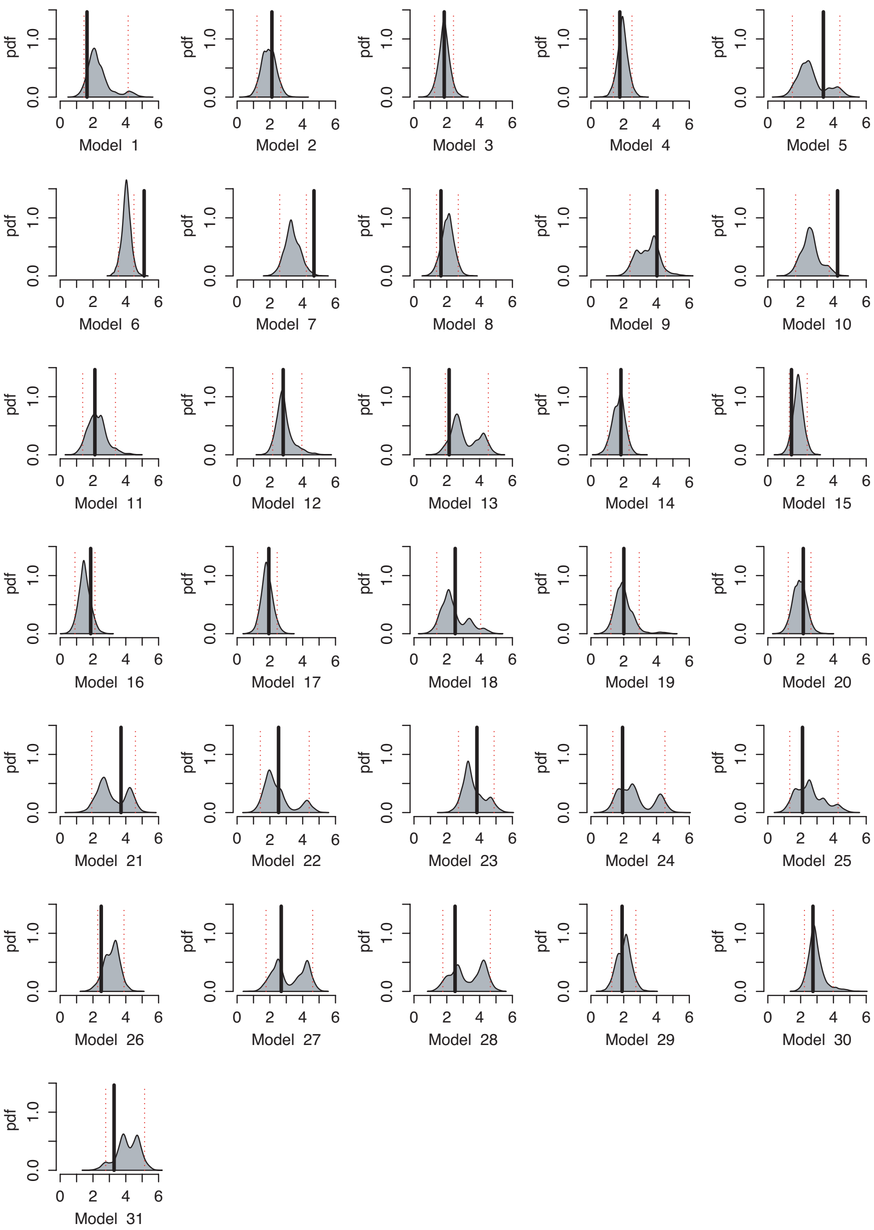

Supplementary Figure 2: **Probability densities from cross-validation with two constraints**. Shown are probability density functions (pdfs) for the global mean surface temperature (GMST) change from preindustrial period [K] needed to melt Arctic sea ice from the observation system simulation experiment using period 1979-2017 for calibration, and both mean sea ice extent (SIE) and SIE sensitivity constraints. Red lines: 90% posterior credible intervals. Black lines: GMST changes from the true models. Each subplot corresponds to a different true climate model. The GMST changes from the true models are within the 90% credible intervals in approximately 9 out of 10 cases, suggesting that the method is well-calibrated.

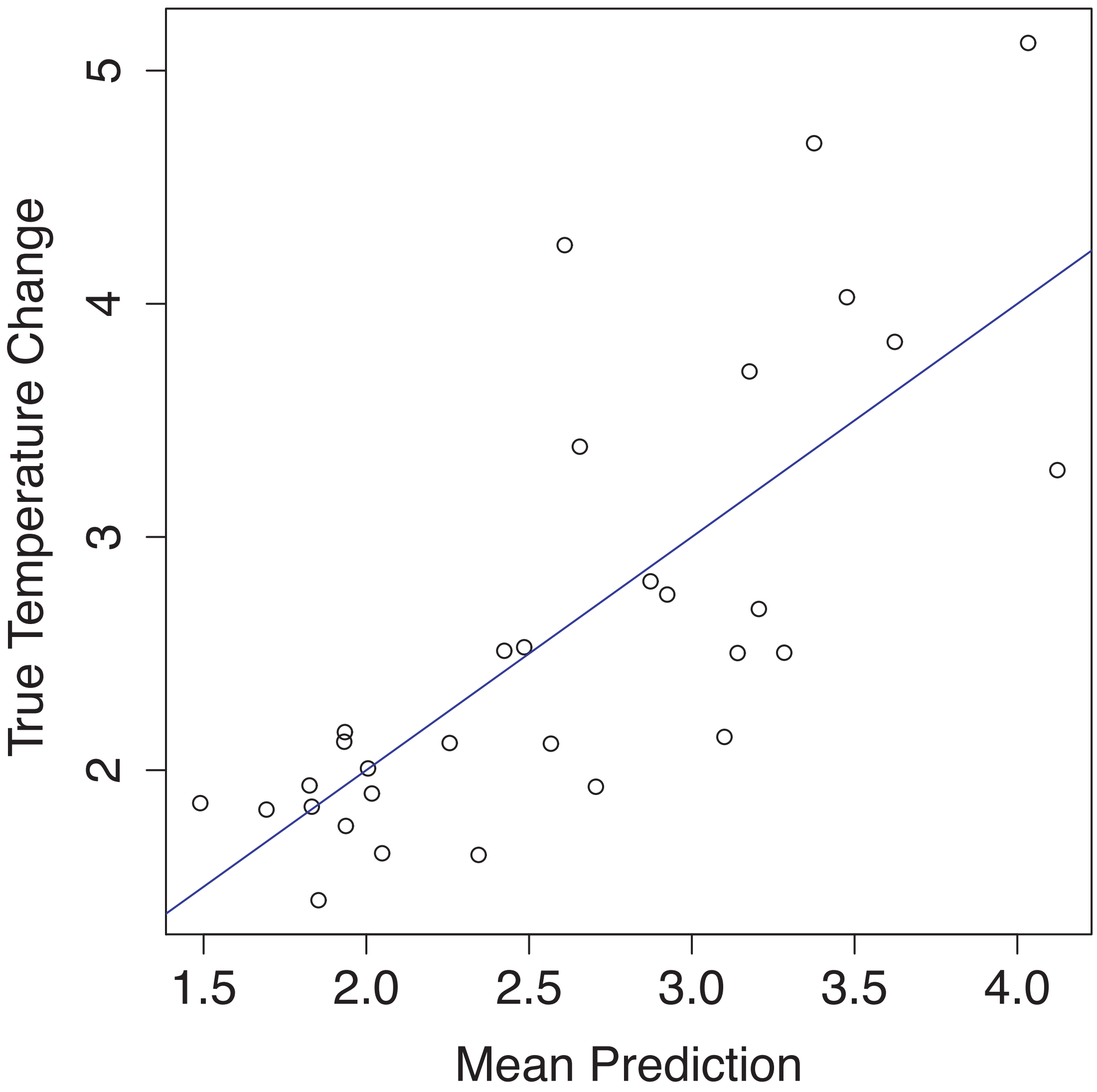

Supplementary Figure 3: **Mean predictions from cross-validation with two constraints.** Scatter plot of global mean temperature change (GMST) change needed to melt Arctic sea ice from the true models (y-axis) [K] vs. the mean prediction from the observation system simulation experiment (x-axis) [K] using period 1979-2017 for calibration, and both mean sea ice extent (SIE) and SIE sensitivity constraints. Blue line is a 1:1 line. This plot shows the skill of the method at projecting GMST change to melt in a perfect-model setting.

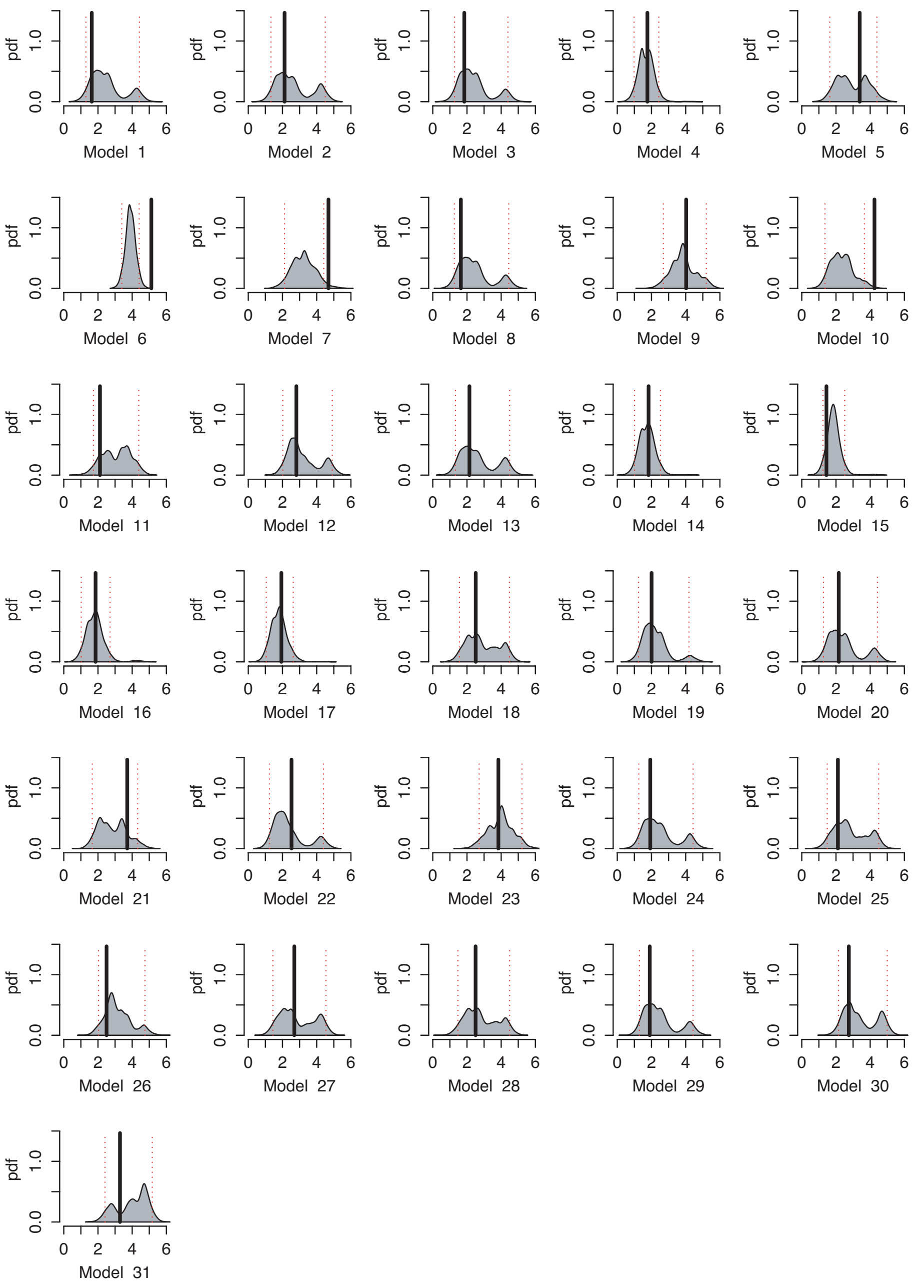

Supplementary Figure 4: **Probability densities from cross-validation with one constraint**. Shown are probability density functions (pdfs) for the global mean surface temperature (GMST) change from preindustrial period [K] needed to melt Arctic sea ice from the observation system simulation experiment using period 1979-2004 for calibration, and only the mean sea ice extent constraint. Red lines: 90% posterior credible intervals. Black lines: GMST changes from the true models. Each subplot corresponds to a different true climate model. The GMST changes from the true models are within the 90% credible intervals in approximately 9 out of 10 cases, suggesting that the method is well-calibrated.

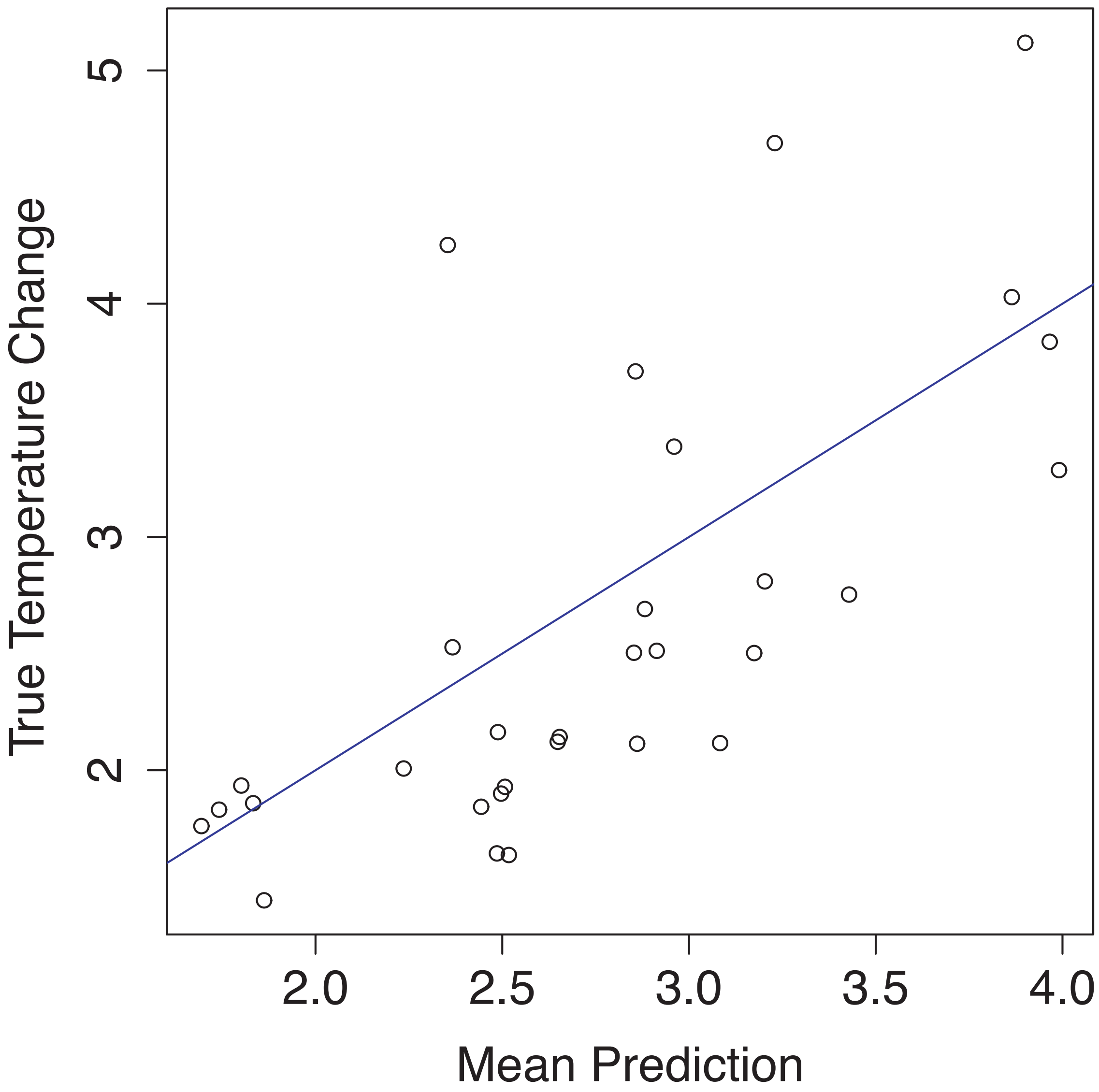

Supplementary Figure 5: **Mean predictions from cross-validation with one constraint.** Scatter plot of global mean temperature change (GMST) change needed to melt Arctic sea ice from the true models (y-axis) [K] vs. the mean prediction from the observation system simulation experiments (x-axis) [K] using period 1979-2004 for calibration, and only the mean sea ice extent constraint. Blue line is a 1:1 line.

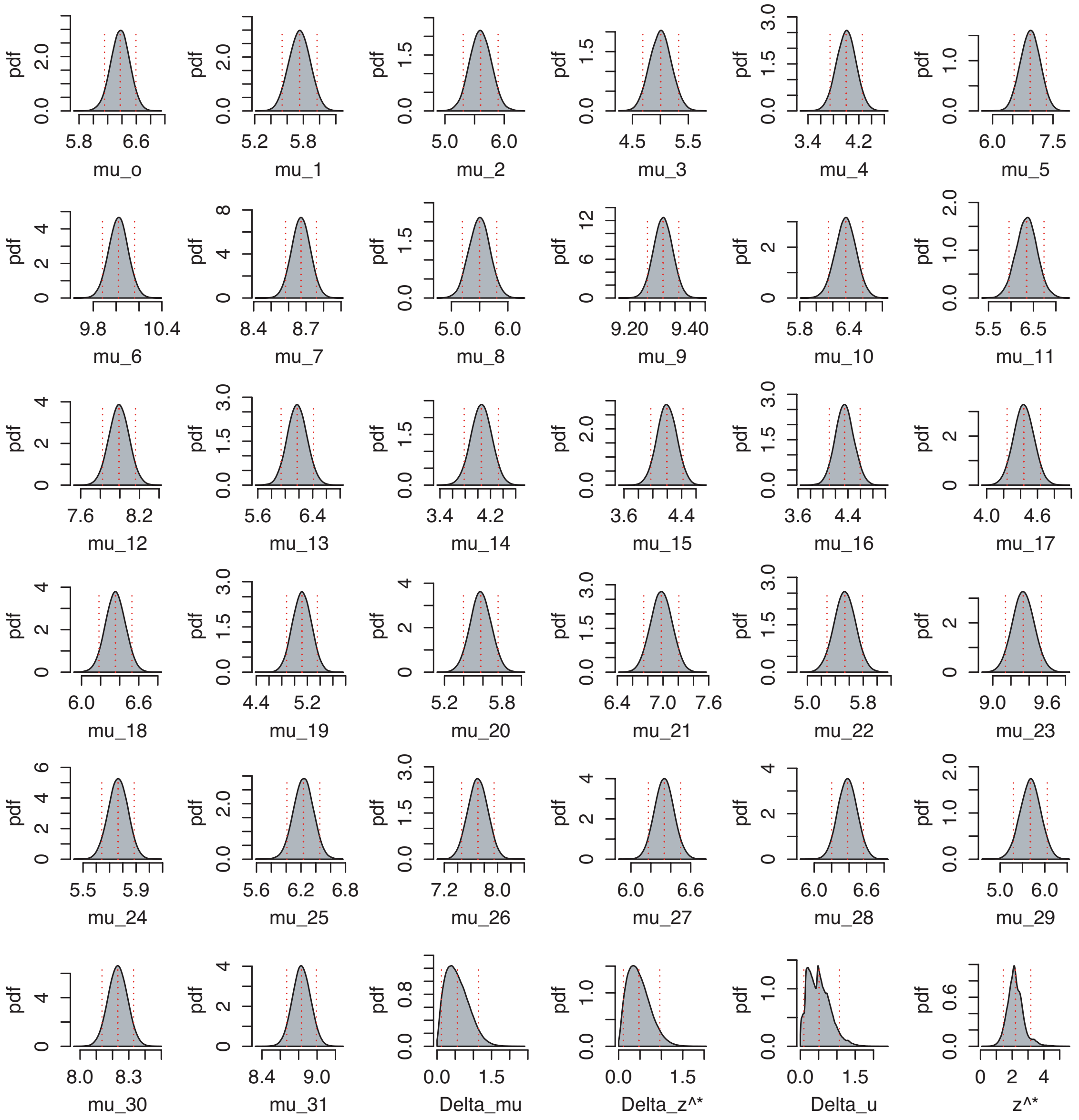

Supplementary Figure 6: **Estimates for all parameters in the HadISST_r51_anthro experiment.** Shown are posterior probability density functions (pdfs) for all statistical model parameters from the HadISST_r51_anthro experiment. Vertical red lines: means and 90% posterior credible intervals. The method jointly estimates pdfs for a large number of parameters using Markov chain Monte Carlo sampling algorithm. One of these parameters is global mean surface temperature change from preindustrial needed to melt Arctic sea ice (z^*). See text for more information about the parameters and/or their units.

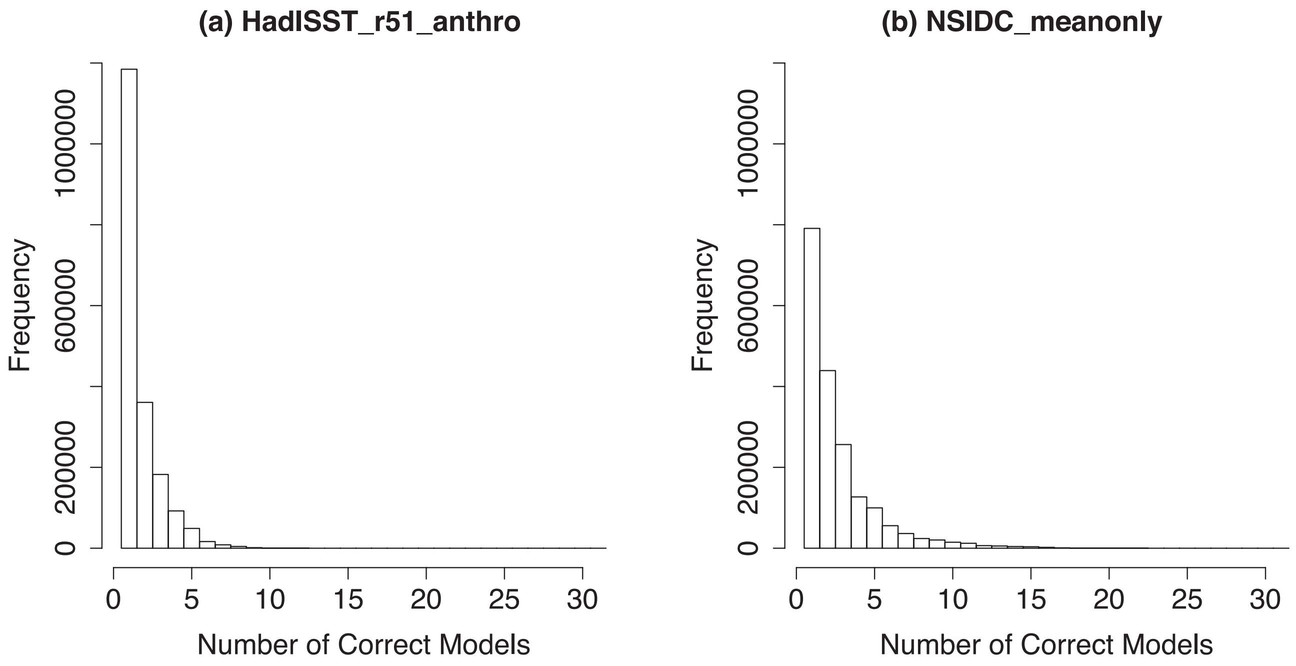

Supplementary Figure 7: **Histograms of the number of correct models**. Shown are histogram (representing relative probabilities) of the number of the correct models from the Markov chain Monte Carlo simulation chains for (a) HadISST_r51_anthro and (b) NSIDC_meanonly experiments. This figure shows that non-exclusive probabilities appear lower once we introduce a second constraint of sea ice sensitivity on the climate models in the HadISST_r51_anthro experiment.

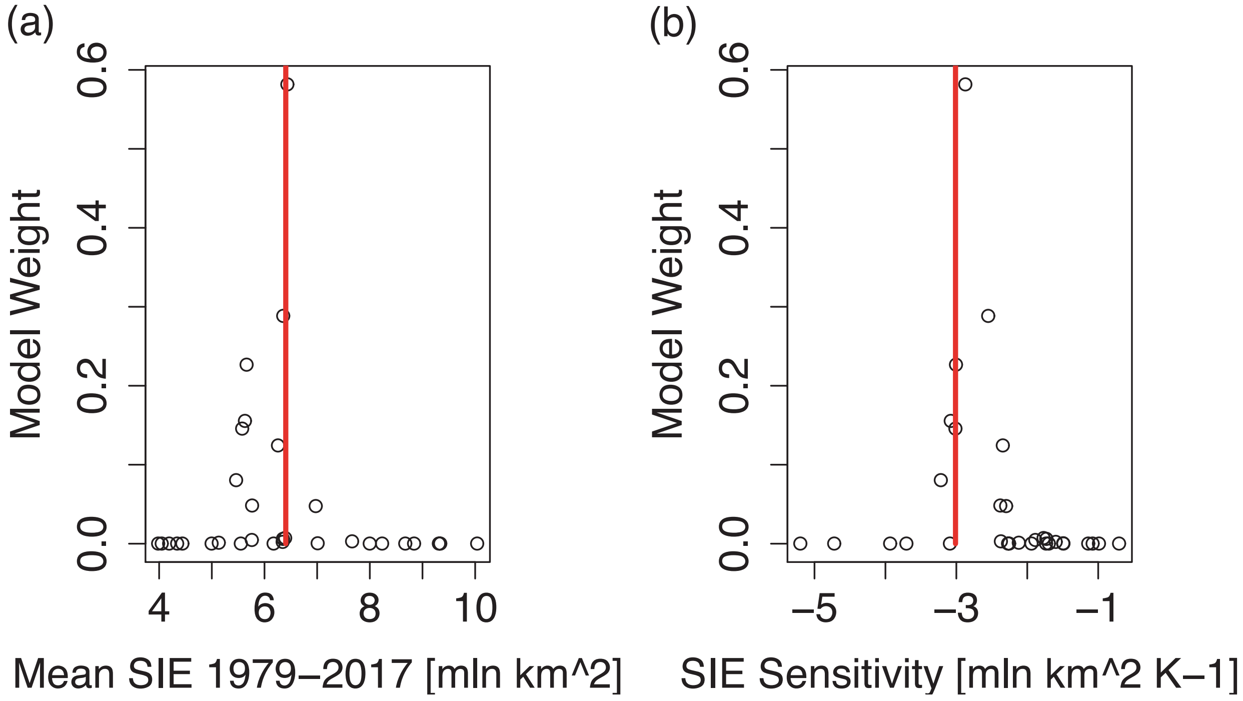

Supplementary Figure 8: **Model weights as a function of model output**. Shown are (a) model weights as a function of mean 1979-2017 sea ice extent (SIE; circles) compared to observations (vertical red line) for the HadISST_r51_anthro experiment, (b) same but for model weights as a function of present-day SIE sensitivity. This figure illustrates that the weights tend to taper off away from the observations. The scatter in the weights is due to the fact that model performance depends on both constraints.

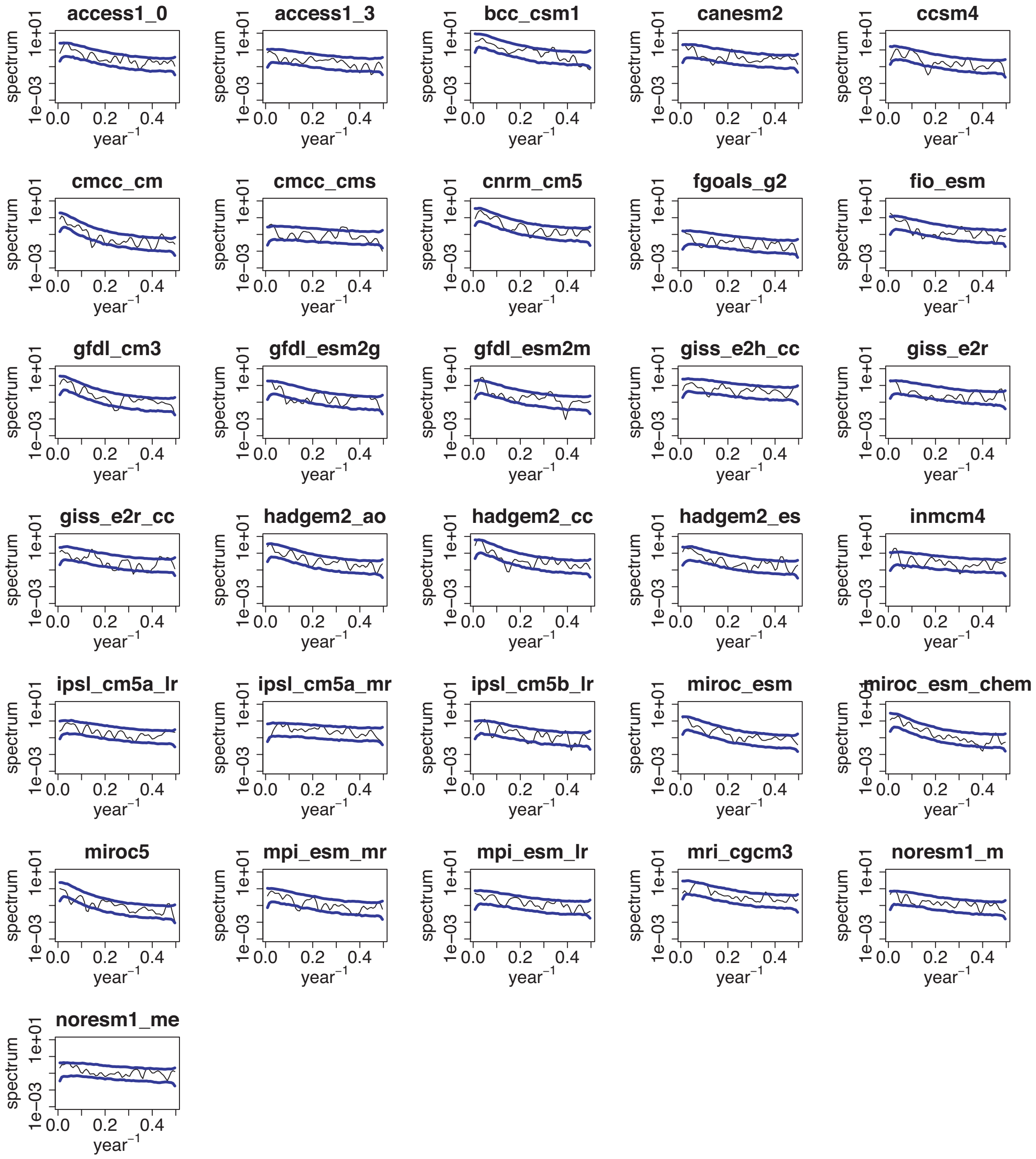

Supplementary Figure 9: **Sea ice extent spectra.** Shown are spectral densities for yearly September Arctic sea ice extent (SIE) model anomalies in climate models from lowess trend lines for the years 1880-2004. Blue lines: 90% confidence intervals for spectra of autoregressive processes of order 1 (AR1) that were fit to modeled anomalies, based on 1000 random realizations for each model. This figure illustrates than an AR1 process appears to be generally reasonable for September SIE.

Supplementary Table 1

Basic information about global climate models that we use.

Model Number	Name	Modeling Centre
1	ACCESS1-0	CSIRO and BOM, Australia
2	ACCESS1-3	CSIRO and BOM, Australia
3	bcc-csm1-1	Beijing Climate Center & China Meteorological Administration, China
4	CanESM2	Canadian Centre for Climate Modeling and Analysis, Canada
5	CCSM4	National Center for Atmospheric Research, USA
6	CMCC-CM	Euro-Mediterranean Centre on Climate Change, Italy
7	CMCC-CMS	Euro-Mediterranean Centre on Climate Change, Italy
8	CNRM-CM5	National Centre for Meteorological Research & European Centre for Research and Advanced Training in Scientific Computation, France
9	FGOALS-g2	Institute of Atmospheric Physics, Chinese Academy of Sciences, China
10	FIO-ESM	The First Institute of Oceanography, SOA, China
11	GFDL-CM3	Geophysical Fluid Dynamics Laboratory, USA
12	GFDL-ESM2G	Geophysical Fluid Dynamics Laboratory, USA
13	GFDL-ESM2M	Geophysical Fluid Dynamics Laboratory, USA
14	GISS-E2-H-CC	NASA Goddard Institute for Space Studies, USA
15	GISS-E2-R	NASA Goddard Institute for Space Studies, USA
16	GISS-E2-R-CC	NASA Goddard Institute for Space Studies, USA
17	HadGEM2-AO	National Institute of Meteorological Research/Korea Meteorological Administration, South Korea

18	HadGEM2-CC	Met Office Hadley Centre, UK
19	HadGEM2-ES	Met Office Hadley Centre, UK
20	INM-CM4	Institute of Numerical Mathematics, Russia
21	IPSL-CM5A-LR	Institut Pierre-Simon Laplace, France
22	IPSL-CM5A-MR	Institut Pierre-Simon Laplace, France
23	IPSL-CM5B-LR	Institut Pierre-Simon Laplace, France
24	MIROC-ESM	University of Tokyo, National Institute for Environmental Studies & Japan Agency for Marine-Earth Science and Technology, Japan
25	MIROC-ESM-CHEM	University of Tokyo, National Institute for Environmental Studies & Japan Agency for Marine-Earth Science and Technology, Japan
26	MIROC5	University of Tokyo, National Institute for Environmental Studies & Japan Agency for Marine-Earth Science and Technology, Japan
27	MPI-ESM-MR	Max Planck Institute for Meteorology (MPI-M), Germany
28	MPI-ESM-LR	Max Planck Institute for Meteorology (MPI-M), Germany
29	MRI-CGCM3	Meteorological Research Institute, Japan
30	NorESM1-M	Norwegian Climate Centre, Norway
31	NorESM1-ME	Norwegian Climate Centre, Norway

Supplementary Note 1

The non-exclusive Law of Total Probability: for arbitrary n events (or n hypotheses) $H_1, H_2, \ldots, H_n$ whose union is S, another event A, and an event corresponding to observing a random variable underlying the dynamical system $\mathbf{Y}$ taking an observed value of $\mathbf{y}$:

$$\begin{aligned} P(A|\mathbf{Y}) = &\sum_{i=1}^{n} P(A|H_i \cap \mathbf{Y})P(H_i|\mathbf{Y}) \\ &- \sum_{1\le i<j\le n} P(A|H_i \cap H_j \cap \mathbf{Y})P(H_i \cap H_j|\mathbf{Y}) \\ &+ \sum_{1\le i<j<k\le n} P(A|H_i \cap H_j \cap H_k \cap \mathbf{Y})P(M_i \cap H_j \cap H_k|\mathbf{Y}) - \cdots \\ &+ (-1)^{n-1}P(A|H_1 \cap H_2 \cap \cdots \cap H_n \cap \mathbf{Y})P(H_1 \cap H_2 \cap \cdots \cap H_n|\mathbf{Y}). \end{aligned} \tag{S1}$$

This law also works for probability density $p(A)$ if A is a continuous random variable. Note that we use capital P for probabilities, and lowercase p for probability densities.

$$\begin{aligned} p(A|\mathbf{Y}) = &\sum_{i=1}^{n} p(A|H_i \cap \mathbf{Y})P(H_i|\mathbf{Y}) \\ &- \sum_{1\le i<j\le n} p(A|H_i \cap H_j \cap \mathbf{Y})P(H_i \cap H_j|\mathbf{Y}) \\ &+ \sum_{1\le i<j<k\le n} p(A|H_i \cap H_j \cap H_k \cap \mathbf{Y})P(H_i \cap H_j \cap H_k|\mathbf{Y}) - \cdots \\ &+ (-1)^{n-1}p(A|H_1 \cap H_2 \cap \cdots \cap H_n \cap \mathbf{Y})P(H_1 \cap H_2 \cap \cdots \cap H_n|\mathbf{Y}). \end{aligned} \tag{S2}$$

Note This equation considers model dependencies, because:

$$\begin{aligned} P(H_1 \cap H_2 \cap \cdots \cap H_n|\mathbf{Y}) = &P(H_1|H_2 \cap \cdots \cap H_n \cap \mathbf{Y})P(H_2|H_3 \cap \cdots H_n \cap \mathbf{Y}) \\ &\cdots P(H_{n-1}|H_n \cap \mathbf{Y})P(H_n|\mathbf{Y}). \end{aligned} \tag{S3}$$

Proof The probability of any event A is formulated as the probability of the event conditional on the proposition that at least one of n hypotheses $H_1, \ldots, H_n$ is correct, *and* that the data $\mathbf{Y} = \mathbf{y}$ have been observed:

$$P(A|\mathbf{Y}) = P(A|[\bigcup_{i=1}^{n} H_i] \cap \mathbf{Y}). \tag{S4}$$

Considering that $P(A|B) = \frac{P(A,B)}{P(B)}$ and that $P(\bigcup_{i=1}^{n} H_i) = 1$, this can be re-written as:

$$\begin{aligned} P(A|\mathbf{Y}) &= \frac{P(A \cap [\bigcup_{i=1}^{n} H_i] \cap \mathbf{Y})}{P([\bigcup_{i=1}^{n} H_i] \cap \mathbf{Y})} = \frac{1}{P(\mathbf{Y})}P(A \cap [\bigcup_{i=1}^{n} H_i] \cap \mathbf{Y}) = \frac{1}{P(\mathbf{Y})}P([A \cap \mathbf{Y}] \cap [\bigcup_{i=1}^{n} H_i]) \\ &= \frac{1}{P(\mathbf{Y})}P(\bigcup_{i=1}^{n}[A \cap H_i \cap \mathbf{Y}]) = \frac{1}{P(\mathbf{Y})}P(\bigcup_{i=1}^{n}[(A \cap \mathbf{Y}) \cap H_i]). \end{aligned} \tag{S5}$$

Now, consider the inclusion-exclusion principle:

$$P(\bigcup_{i=1}^{n} A) = P(A_1 \cup A_2 \cup \cdots \cup A_n) = \sum_i P(A_i) - \sum_{1 \leq i < j \leq n} P(A_i \cap A_j)$$
$$+ \sum_{1 \leq i < j < k \leq n} P(A_i \cap A_j \cap A_k) - \cdots \tag{S6}$$
$$+ (-1)^{n-1} P(A_1 \cap A_2 \cap \cdots \cap A_n).$$

Then:

$$\begin{aligned}
P(A|\mathbf{Y}) &= \frac{1}{P(\mathbf{Y})} P(\bigcup_{i=1}^{n} [(A \cap \mathbf{Y}) \cap H_i]) \\
&= \frac{1}{P(\mathbf{Y})} \sum_{i=1}^{n} P(A \cap H_i \cap \mathbf{Y}) \\
&\quad - \frac{1}{P(\mathbf{Y})} \sum_{1 \leq i < j \leq n} P(([A \cap \mathbf{Y}] \cap H_i) \cap ([A \cap \mathbf{Y}] \cap H_j)) \\
&\quad + \frac{1}{P(\mathbf{Y})} \sum_{1 \leq i < j < k \leq n} P(([A \cap \mathbf{Y}] \cap H_i) \cap ([A \cap \mathbf{Y}] \cap H_j) \cap ([A \cap \mathbf{Y}] \cap H_k)) - \cdots \\
&\quad + \frac{1}{P(\mathbf{Y})} (-1)^{n-1} P(([A \cap \mathbf{Y}] \cap H_1) \cap ([A \cap \mathbf{Y}] \cap H_2) \cap \cdots \cap ([A \cap \mathbf{Y}] \cap H_n)) \\
&= \frac{1}{P(\mathbf{Y})} \sum_{i=1}^{n} P(A|H_i \cap \mathbf{Y}) P(H_i|\mathbf{Y}) P(\mathbf{Y}) \\
&\quad - \frac{1}{P(\mathbf{Y})} \sum_{1 \leq i < j \leq n} P(A|H_i \cap H_j \cap \mathbf{Y}) P(H_i \cap H_j|\mathbf{Y}) P(\mathbf{Y}) \\
&\quad + \frac{1}{P(\mathbf{Y})} \sum_{1 \leq i < j < k \leq n} P(A|H_i \cap H_j \cap H_k \cap \mathbf{Y}) P(H_i \cap H_j \cap H_k|\mathbf{Y}) P(\mathbf{Y}) - \cdots \\
&\quad + \frac{1}{P(\mathbf{Y})} (-1)^{n-1} P(A|H_1 \cap H_2 \cap \cdots \cap H_n \cap \mathbf{Y}) P(H_1 \cap H_2 \cap \cdots \cap H_n|\mathbf{Y}) P(\mathbf{Y}), \\
&= \sum_{i=1}^{n} P(A|H_i \cap \mathbf{Y}) P(H_i|\mathbf{Y}) \\
&\quad - \sum_{1 \leq i < j \leq n} P(A|H_i \cap H_j \cap \mathbf{Y}) P(H_i \cap H_j|\mathbf{Y}) \\
&\quad + \sum_{1 \leq i < j < k \leq n} P(A|H_i \cap H_j \cap H_k \cap \mathbf{Y}) P(H_i \cap H_j \cap H_k|\mathbf{Y}) - \cdots \\
&\quad + (-1)^{n-1} P(A|H_1 \cap H_2 \cap \cdots \cap H_n \cap \mathbf{Y}) P(H_1 \cap H_2 \cap \cdots \cap H_n|\mathbf{Y}),
\end{aligned}$$

which is the discrete version of the law. For a continuous random variable A we define [63]:

$$p_a(A) = p(A) = \lim_{\delta a \to 0} \frac{P(a \leq A < a + \delta a)}{\delta a} \tag{S7}$$

and by analogy:

$$p_a(A|H) = p(A|H) = \lim_{\delta a \to 0} \frac{P(a \leq A < a + \delta a|H)}{\delta a}. \tag{S8}$$

We first rewrite equation (S1) as:

$$
\begin{aligned}
P(a \le A < a+\delta a|\mathbf{Y}) = &\sum_{i=1}^{n} P(a \le A < a+\delta a|H_i \cap \mathbf{Y})P(H_i|\mathbf{Y}) \\
&- \sum_{1\le i<j\le n} P(a \le A < a+\delta a|H_i \cap H_j \cap \mathbf{Y})P(H_i \cap H_j|\mathbf{Y}) \\
&+ \sum_{1\le i<j<k\le n} P(a \le A < a+\delta a|H_i \cap H_j \cap H_k \cap \mathbf{Y})P(M_i \cap H_j \cap H_k|\mathbf{Y}) - \cdots \\
&+ (-1)^{n-1} P(a \le A < a+\delta a|H_1 \cap H_2 \cap \cdots \cap H_n \cap \mathbf{Y})P(H_1 \cap H_2 \cap \cdots \cap H_n|\mathbf{Y}).
\end{aligned} \tag{S9}
$$

Now we divide both sides by δa:

$$
\begin{aligned}
\frac{P(a \le A < a+\delta a|\mathbf{Y})}{\delta a} = &\sum_{i=1}^{n} \frac{P(a \le A < a+\delta a|H_i \cap \mathbf{Y})}{\delta a} P(H_i|\mathbf{Y}) \\
&- \sum_{1\le i<j\le n} \frac{P(a \le A < a+\delta a|H_i \cap H_j \cap \mathbf{Y})}{\delta a} P(H_i \cap H_j|\mathbf{Y}) \\
&+ \sum_{1\le i<j<k\le n} \frac{P(a \le A < a+\delta a|H_i \cap H_j \cap H_k \cap \mathbf{Y})}{\delta a} P(H_i \cap H_j \cap H_k|\mathbf{Y}) - \cdots \\
&+ (-1)^{n-1} \frac{P(a \le A < a+\delta a|H_1 \cap H_2 \cap \cdots \cap H_n \cap \mathbf{Y})}{\delta a} P(H_1 \cap H_2 \cap \cdots \cap H_n|\mathbf{Y}),
\end{aligned} \tag{S10}
$$

and take a limit:

$$
\begin{aligned}
\lim_{\delta a \to 0} \frac{P(a \le A < a+\delta a|\mathbf{Y})}{\delta a} = &\sum_{i=1}^{n} \lim_{\delta a \to 0} \left[\frac{P(a \le A < a+\delta a|H_i \cap \mathbf{Y})}{\delta a}\right] P(H_i|\mathbf{Y}) \\
&- \sum_{1\le i<j\le n} \lim_{\delta a \to 0} \left[\frac{P(a \le A < a+\delta a|H_i \cap H_j \cap \mathbf{Y})}{\delta a}\right] P(H_i \cap H_j|\mathbf{Y}) \\
&+ \sum_{1\le i<j<k\le n} \lim_{\delta a \to 0} \left[\frac{P(a \le A < a+\delta a|H_i \cap H_j \cap H_k \cap \mathbf{Y})}{\delta a}\right] P(H_i \cap H_j \cap H_k|\mathbf{Y}) - \cdots \\
&+ (-1)^{n-1} \lim_{\delta a \to 0} \left[\frac{P(a \le A < a+\delta a|H_1 \cap H_2 \cap \cdots \cap H_n \cap \mathbf{Y})}{\delta a}\right] P(H_1 \cap H_2 \cap \cdots \cap H_n|\mathbf{Y}).
\end{aligned} \tag{S11}
$$

Substituting results from Equations (S7) and (S8) gives the continuous version of the law:

$$
\begin{aligned}
p(A|\mathbf{Y}) = &\sum_{i=1}^{n} p(A|H_i \cap \mathbf{Y})P(H_i|\mathbf{Y}) \\
&- \sum_{1\le i<j\le n} p(A|H_i \cap H_j \cap \mathbf{Y})P(H_i \cap H_j|\mathbf{Y}) \\
&+ \sum_{1\le i<j<k\le n} p(A|H_i \cap H_j \cap H_k \cap \mathbf{Y})P(H_i \cap H_j \cap H_k|\mathbf{Y}) - \cdots \\
&+ (-1)^{n-1} p(A|H_1 \cap H_2 \cap \cdots \cap H_n \cap \mathbf{Y})P(H_1 \cap H_2 \cap \cdots \cap H_n|\mathbf{Y}).
\end{aligned} \tag{S12}
$$

Supplementary Note 2

In this section we describe how our framework can be applied to the previous work [11]. They use SVD and MDS techniques to map present-day spatial model output and observations into

a 2D space. Thus, associated with each model i are deterministic values of $\mathbf{R}_i = (R_{i,1}, R_{i,2})$, and observations are represented by a value $\mathbf{Y} = (Y_1, Y_2)$. Associated with each model is also deterministic climate sensitivity CS_i output. They then use deterministic interpolation to interpolate modeled climate sensitivity to any parameter value $\mathbf{R}$ [11]. Effectively, they assume the following statistical model in their Gaussian prior experiment:

$$\mathbf{Y} \sim N(R_1, R_2, \sigma_1, \sigma_2, \rho = 0). \tag{S13}$$

The observations come from an uncorrelated bivariate normal distribution centered on some true unknown random parameter value $\mathbf{R} = (R_1, R_2)$, with known standard deviations σ_1 and σ_2 set to standard deviations of model ensemble. The distribution reflects the uncertainty in observations due to internal variability and observational error. It is beyond the scope of this work to justify this statistical model. They only consider a region within the convex hull of the models, which can be regarded as a crude approximation to the sample space of the union of all models $S = \bigcup H_i$ [11]. (There are some issues with such representation: for example, if a particular model is correct, then values slightly outside of the convex hull close to this model should also be acceptable. Also, if there is a large gap within the convex hull without any models, such region should be discarded from the sample space. Nonetheless, the convex hull can be thought of a crude attempt to delineate the probability space). Effectively, this means the following prior for parameters: $p(\mathbf{R}) = 1_S$, where 1_S is defined as in the main text. Under such a model the posterior probability of the true climate parameters $\mathbf{R}$ given the observations is (using the Bayes theorem):

$$\begin{aligned} p(\mathbf{R}|\mathbf{Y}) \propto & p(\mathbf{Y}|\mathbf{R})p(\mathbf{R}) \\ =& \frac{1}{2\pi\sigma_1\sigma_2} \exp\left(-\frac{1}{2}\left[\left(\frac{Y_1 - R_1}{\sigma_1}\right)^2 + \left(\frac{Y_2 - R_2}{\sigma_2}\right)^2\right]\right) 1_S \\ =& \frac{1}{2\pi\sigma_1\sigma_2} \exp\left(-\frac{1}{2}\left[\left(\frac{R_1 - Y_1}{\sigma_1}\right)^2 + \left(\frac{R_2 - Y_2}{\sigma_2}\right)^2\right]\right) 1_S \\ =& N(Y_1, Y_2, \sigma_1, \sigma_2, \rho = 0) 1_S, \end{aligned} \tag{S14}$$

where $p(\mathbf{Y}|\mathbf{R})$ is the bivariate normal likelihood of the observations. The result is a bivariate normal distribution centered on the observations, with standard deviations equal to the model ensemble standard deviations, and truncated outside of the sample space. Associated with each value of $\mathbf{R}$ is the interpolated value of climate sensitivity CS: it is deterministic but can be represented using a degenerate distribution $p(CS|\mathbf{R})$. In this case, the joint distribution of climate sensitivity and present-day climate parameters is defined by:

$$p(CS, \mathbf{R}|\mathbf{Y}) = \frac{1}{p(\mathbf{Y})} p(CS, \mathbf{R}, \mathbf{Y}) = p(CS|\mathbf{R}) p(\mathbf{R}|\mathbf{Y}). \tag{S15}$$

The study then samples from this joint distribution, and looks at the marginal distribution for CS, which follows the marginalization theorem in Equation (8) in the main text:

$$p(CS|\mathbf{Y}) = \int p(CS, \mathbf{R}|\mathbf{Y}) d\mathbf{R} = \int p(CS|\mathbf{R}) p(\mathbf{R}|\mathbf{Y}) d\mathbf{R}. \tag{S16}$$

As we show in Equation (7) in the main text, this approach (albeit relying on crude statistical assumptions) considers *all* model interactions of the order n (where n is the number of models):

$$\begin{aligned}
p(CS|\mathbf{Y}) = \int p(CS, \mathbf{R}|\mathbf{Y}) d\mathbf{R} = \int p(CS|\mathbf{R}) p(\mathbf{R}|\mathbf{Y}) d\mathbf{R} \\
= \sum_{i=1}^{n} p(CS|H_i \cap \mathbf{Y}) P(H_i|\mathbf{Y}) \\
- \sum_{1 \le i < j \le n} p(CS|H_i \cap H_j \cap \mathbf{Y}) P(H_i \cap H_j|\mathbf{Y}) \\
+ \sum_{1 \le i < j < k \le n} p(CS|H_i \cap H_j \cap H_k \cap \mathbf{Y}) P(H_i \cap H_j \cap H_k|\mathbf{Y}) - \cdots \\
+ (-1)^{n-1} p(CS|H_1 \cap H_2 \cap \cdots \cap H_n \cap \mathbf{Y}) P(H_1 \cap H_2 \cap \cdots \cap H_n|\mathbf{Y}).
\end{aligned} \tag{S17}$$

Supplementary References

[63] *Bayesian Logical Data Analysis for the Physical Sciences.* Cambridge University Press, Cambridge, UK, 2005.
