# Supplementary figures and images for: A novel method to test non-exclusive hypotheses applied to Arctic ice projections from dependent models
Source: Nat Commun. 2019 Jul 9;10:3016. doi: 10.1038/s41467-019-10561-x (PMC6616623; doi:10.1038/s41467-019-10561-x)

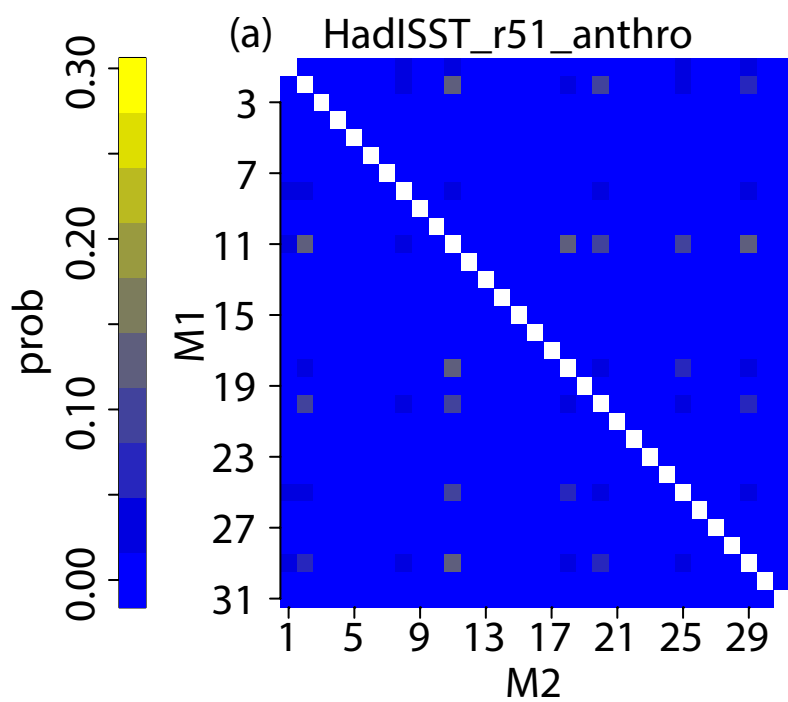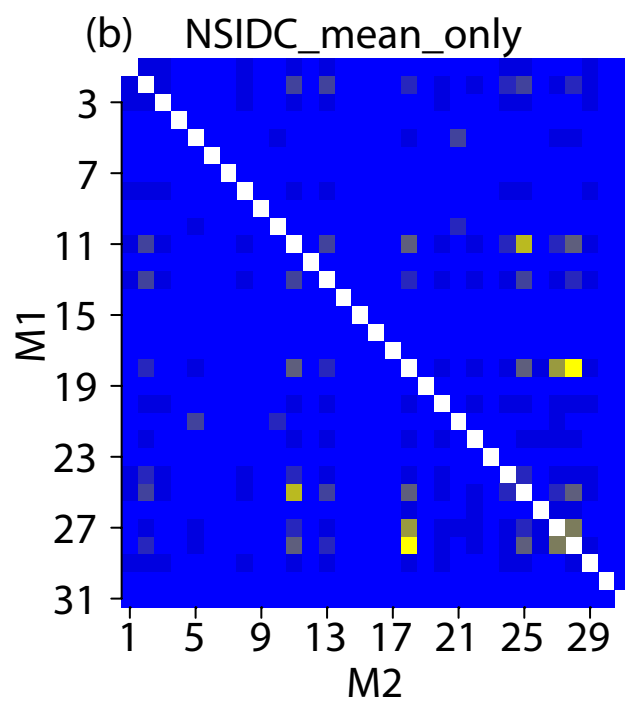

Supplement: Supplementary file 3 — Figure 2 [file 41467_2019_10561_MOESM3_ESM.pdf]
